# Talonavicular joint mobilization and foot core strengthening in patellofemoral pain syndrome: a single-blind, three-armed randomized controlled trial

**DOI:** 10.1186/s12891-022-05099-x

**Published:** 2022-02-15

**Authors:** Hyun-Joong Kim, Juchul Cho, Seungwon Lee

**Affiliations:** 1 Department of Physical Therapy, The Graduate School of Sahmyook University, Seoul, Republic of Korea; 2 Department of Rehabilitation Center, Wellcity Hospital, Daejeon, Republic of Korea; 3 Department of Physical Therapy, Sahmyook University, 815 Hwarang-ro, Nowon-gu, 01795 Seoul, Republic of Korea

**Keywords:** Patellofemoral pain syndrome, Flat foot, Manual therapy, Physical therapy, Pain, Recovery of function

## Abstract

**Background:**

Patellofemoral pain syndrome (PFPS) is defined as pain around the patella while performing activities such as squats, running, and climbing steps. One of the inherent risk factors for PFPS is an excessively pronated foot posture. The aim of this study was to investigate the effect of foot intervention, talonavicular joint mobilization (TJM) and foot core strengthening (FCS), on PFPS.

**Methods:**

Forty-eight patients with PFPS (mean age, 21.96 ± 2.34 years; BMI, 22.77 ± 2.95 kg/m^2^) were enrolled in the study. Participants were randomly assigned in a 1:1:1 ratio to three groups, and received 12 sessions of TJM, FCS, and blended intervention at university laboratory for 4 weeks. The primary outcomes were pain while the secondary outcomes were lower extremity function, valgus knee, foot posture, and muscle activity ratio measured at baseline, after 12 sessions, and at the 4-week follow-up.

**Results:**

The two-way repeated-measures ANOVA revealed significant interactions in all groups (*p* < 0.05). TJM reduced pain more than the FCS at post-test (mean difference, − 0.938; 95% Confidence interval [CI], − 1.664 to − 0.211; *p* < 0.05), and blended intervention improved lower extremity function (mean difference, 6.250; 95% CI, 1.265 to 11.235; *p* < 0.05) and valgus knee (mean difference, − 11.019; 95% CI, − 17.007 to − 5.031; *p* < 0.05) more than the TJM at 4 weeks follow-up. TJM was more effective in post-test (mean difference, − 1.250; 95% CI, − 2.195 to − 0.305; *p* < 0.05), and TJM (mean difference, − 1.563; 95% CI, − 2.640 to − 0.485; *p* < 0.05) and blended intervention (mean difference, − 1.500; 95% CI, − 2.578 to − 0.422; *p* < 0.05) were more effective in foot posture than the FCS in 4 weeks follow-up. Blended intervention displayed greater improvement in muscle activity than the TJM (mean difference, 0.284; 95% CI, 0.069 to 0.500; *p* < 0.05) and the FCS (mean difference, 0.265; 95% CI, 0.050 to 0.481; *p* < 0.05) at 4 weeks follow-up.

**Conclusions:**

Our study is a novel approach to the potential impact of foot interventions on patellofemoral pain. Foot intervention including TJM and FCS is effective for pain control and function improvement in individuals with PFPS.

**Trial registration:**

KCT0003176, 16/08/2018 (retrospectively registered).

**Supplementary Information:**

The online version contains supplementary material available at 10.1186/s12891-022-05099-x.

## Background

Patellofemoral pain syndrome (PFPS) is defined as pain around the patella due to activities (squats, running, climbing, etc.) that load the patellofemoral joint without pathological changes [1, 2]. It is a common problem among adolescents and young adults, and the annual prevalence of patellofemoral pain in the general population was reported to be 22.7 and 28.9% in adolescents [3–5].

The rehabilitation of PFPS includes conservative treatments for symptoms such as muscle strengthening exercises (quadriceps femoris and hip abductors), flexibility exercises (rectus femoris, hamstring, gastrocnemius, hip flexors), foot orthoses to reduce pronated foot, patellofemoral joint taping, braces, and non-steroidal anti-inflammatory drugs (NSAIDs) [2, 6]. Some studies have reported a pronated foot as an intrinsic risk factor for PFPS, suggesting that it may be a solution to the underlying problem [7].

Abnormal motion of the talonavicular joint due to navicular drop or drift in the pronated foot is an indicator of the overall function of the foot [8]. Barton et al. reported an excessively pronated foot in patients with PFPS [9]. Similarly, studies have shown a correlation between the pronated foot and PFPS [10–12]. A pronated foot is defined as the flattening or loss of the medial longitudinal arch (MLA) [13, 14]. Changes in the lower extremity alignment can cause calcaneal eversion, tibia internal rotation, valgus knee, and femur internal rotation in the normal structure, altering the angle of muscle contraction of the quadriceps femoris, causing the patella to track in the lateral direction, resulting in lower extremity dysfunction [15, 16].

Foot interventions in patients with patellofemoral pain have shown significant effectiveness of foot orthoses in addressing hypermobility of the talonavicular joint [7]. A study compared to a group that only performed knee joint exercise for 12 weeks also found that foot orthoses was effective [17]. These findings suggest that therapeutic interventions for the foot, an intrinsic risk factor, can address the fundamental component of knee pain. However, interventions for pronated foot are mainly limited to foot orthoses and there is a lack of related research.

In this study, foot core strengthening (FCS) [18] were performed to maintain foot posture through MLA support and talonavicular joint mobilization (TJM) [19, 20] to control hypermobility of the talonavicular joint of the pronated foot. We also compared the persistence of the effects of each intervention through blended intervention with TJM and FCS. Thus, this study aimed to investigate the effect of TJM and FCS applied to the pronated foot on PFPS. The hypothesis in this study is that improvement of the pronated foot through foot interventions will cause changes in PFPS pain, lower extremity function, valgus knee, foot posture, and muscle activity rate, and there will be differences for each foot intervention.

## Methods

### Study design

This single-blind, three-group, parallel-arm, randomized controlled trial included three evaluation sessions (baseline, post-test, and follow-up at 4 weeks) and a four-week intervention (TJM, FCS, or blended intervention). The primary outcomes were pain (numeric pain rating scale [NPRS]) and lower extremity function (anterior knee pain scale [AKPS]), while the secondary outcomes were valgus knee (dynamic valgus index [DVI]), foot posture (foot posture index [FPI]), and muscle activity ratio (vastus medialis/vastus lateralis [VM/VL] muscle activity ratio).

### Participants

This study recruited 86 men and women in their twenties with unilateral anterior knee pain as potential participants through the bulletin board of Gwangju Health University in Gwangju Metropolitan City, South Korea. Thirty-eight participants who did not satisfy the study inclusion criteria were excluded by the physical therapist (H.J.). Forty-eight participants satisfying the criteria were informed about the study’s details and purpose and agreed to participate in the study. Table 1 shows the inclusion and exclusion criteria [17, 21–25]. This study protocol was approved by the institutional review board of Sahmyook University (2–7,001,793-AB-N-012018055HR). This randomized controlled trial was registered in the Clinical Research Information Service (KCT0003176). Data were collected at the Physical Therapy Diagnostic Laboratory, Gwangju Health University.

**Table 1:** Inclusion and exclusion criteria

Inclusion Criteria	Exclusion Criteria
• Anterior or posterior pain in the knee area lasting for more than 12 weeks • Excessive calcaneal eversion measured at 6° in the relaxed posture • Results for at least two of the following four tests (isometric contraction during slight knee flexion, palpation of the knee joint line, compression of the knee bone against the femur, and actively limited knee extension) • Score of between three and seven points on the numerical pain rating scale during the last week’s activities of daily living	• A history of diagnosis of meniscus or joint injury • Cruciate or collateral laxity or tenderness • Patellar tendon, iliotibial band, or pes anserine tenderness • Signs of patellar apprehension test positivity • Osgood–Schlatter or Sind–Larsen–Johansson syndrome • Traces of effusion • Referred hip or back pain • History of recurrent knee joint subluxation or dislocation • History of knee joint surgery • Taking nonsteroidal anti-inflammatory drugs or corticosteroids within 24 h before the test • History of brain injury or vestibular disorder within the last 6 months • Pregnant woman

### Randomization and blinding

The participants were randomly assigned in a 1:1:1 ratio to each TJM (experimental group 1), FCS (control group), and blended intervention group (experimental group 2) using random allocation software (Isfahan University, Iran). The block setting was three equal sizes and the code for identification was randomly generated with four digits. The intervention schedules were planned according to groups receiving the same intervention to blind participants. The assessor (J.C.) remained the same throughout the study and the therapist was not blinded.

### Intervention

After randomization, training was conducted on the evaluation procedure and the progress of the intervention program before each intervention and evaluation to minimize errors during the experiment. Participants were asked to receive interventions three times a week for four weeks and participate in each intervention according to the set schedules. Participants were asked to engage in activities as usual and not receive other interventions regarding feet during the experiment.

#### Talonavicular joint mobilization (TJM)

TJM is a grade III maitland technique and is applied with a high amplitude from the end range and 1 s of vibration in the middle range of the joint through a linear motion to where tissue resistance is felt [21, 26, 27]. In the prone position, the patient is supported by a towel placed under the foot. The therapist’s fixing hand wraps the calcaneus, grasps the talus bone, and fixes it [19, 20]. The other hand holds the navicular and glides in the dorsal direction. Two sets of 5 min total were performed for 4 weeks (Additional file 1) [24].

#### Foot core strengthening (FCS)

FCS is an exercise that effectively mobilizes the abductor hallucis and prevents excessive pronation of the MLA [28–30]. FCS repeats isometric contraction for 5 s by pulling the metatarsal head towards the heel using an intrinsic foot muscle [30]. Two sets are performed for a total of 5 min. For weeks 1–2 weeks, the participants were seated in a chair; for weeks 3–4, they were in a standing position (Additional file 2) [31, 32].

#### Blended intervention

A blended intervention with TJM and FCS was performed for 4 weeks. The intervention comprised one set of TJM and one set of FCS for a total of 5 min [24, 30].

### Outcomes

A total of 12 sessions were performed in a university laboratory for 4 weeks. The participants were followed up at 4 weeks after each intervention.

#### Primary outcome measures

The primary outcome was the 11-point NPRS to measure pain intensity. The NPRS defines pain intensity from 0 to 10 points, with 0 and 10 points indicating no pain and the most severe pain imaginable, respectively [33]. NPRS measurements assessed the intensity of pain experienced in the past 24 h. The reported test-retest reliability (intraclass correlation coefficient [ICC]) was 0.76, demonstrating it to be a good indicator of pain intensity, with a minimum clinically important difference (MCID) score of 2 points [34, 35].

#### Secondary outcome measures

The secondary outcomes in the present study included the AKPS, DVI, FPI, and VM/VL muscle activity ratio. The AKPS, known as the Kujala scale, is a self-reported questionnaire for knee joints that contains 13 different items and a maximum score of 100 points, with higher scores indicating better lower extremity function [36]. This scale is mainly recommended for patients presenting symptoms of PFPS, and the test-retest reliability ICC was 0.95 (MCID: 13 points) [7, 26, 35, 37].

**Fig. 1:**
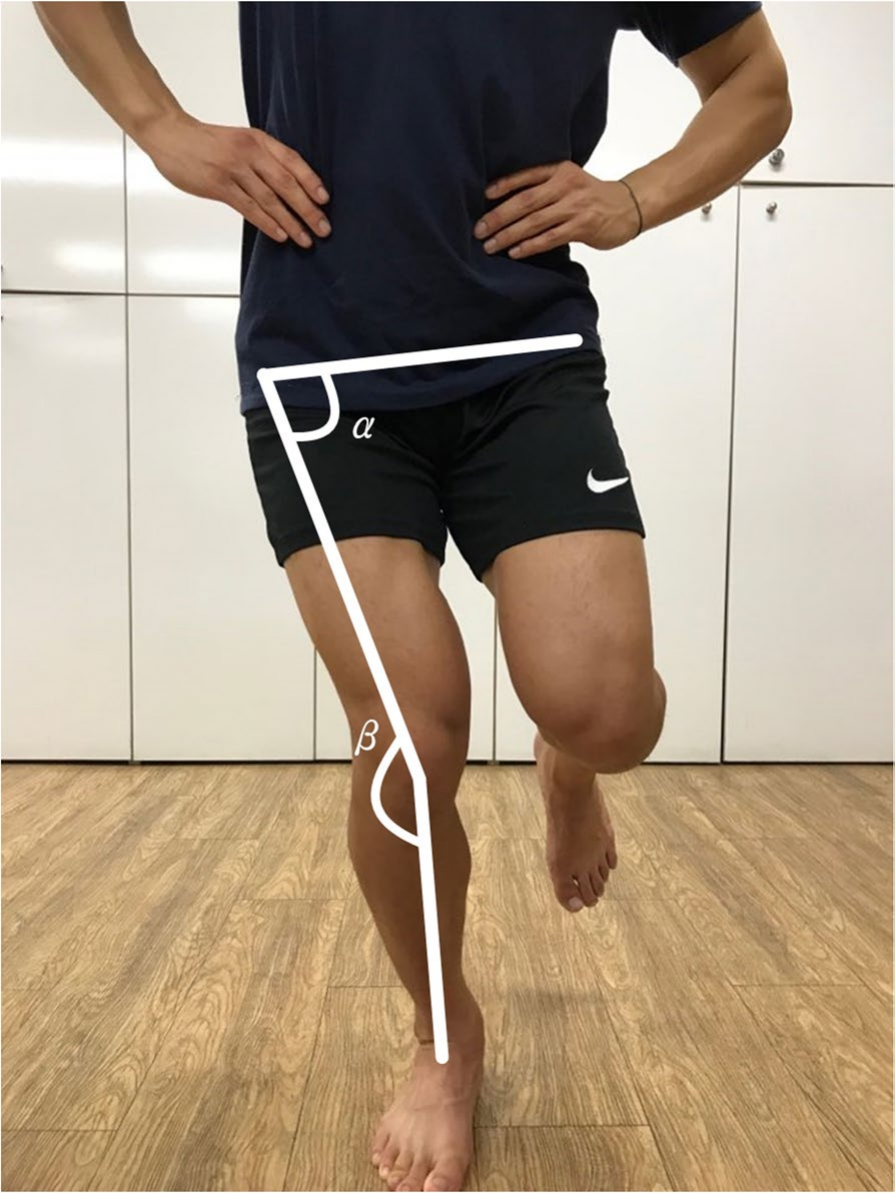
Dynamic valgus index (DVI). The DVI is calculated as the sum of the frontal plane projection angle (FPPA) of the knee joint and the hip FPPA. **A** Hip FPPA: 90° minus the angle (α) between the pelvic part (the line connecting both anterior superior iliac spine [ASIS]) and the thigh (the line connecting the midpoint of the patella at the ASIS). **B** Knee FPPA: 180° minus the angle (β) between the thigh and the shin (the line connecting the midpoint of the patella and the midpoint of the ankle)

Dynamic knee valgus was measured using the DVI and defined as a knee projection angle increase of 10° or more during flexion of a leg in a single-leg squat [36, 38]. The DVI is calculated as the sum of the frontal plane projection angle (FPPA) of the knee joint and the hip FPPA. The hip FPPA is calculated as 90° minus the angle (α) between the pelvic part (the line connecting both anterior superior iliac spine [ASIS]) and the thigh (the line connecting the midpoint of the patella at the ASIS). In addition, the knee joint FPPA was calculated by subtracting the angle (β) between the thigh and shin (the line connecting the midpoint of the patella and the midpoint of the ankle) at 180° [38]. The measurements were performed using a mobile phone camera (iPhone 7 plus, Apple, USA, 2016) for the collection of two-dimensional data, with the camera placed 3 m in front of the participants at a height of 45 cm. Data analysis was performed using image analysis software (ProSuite 5.5, DARTFISH, Switzerland, 2006). Two-dimensional motion analysis is a common measurement tool because it is less expensive and more effective than three-dimensional motion analysis and is easily accessible for use in the clinic. The reported reliability of measurement (ICC) is 0.68–0.83 [17, 21, 24, 25]. To minimize measurement errors, the same inspector performed measurements at baseline, post-intervention, and at the 4-week follow-up (Fig. 1).

The FPI is a foot posture assessment tool comprising six items (talar head palpation, curves above and below the lateral malleoli, calcaneal angle, talonavicular bulge, medial longitudinal arch, and forefoot to rearfoot alignment), with each item scored from − 2 to + 2 points (− 12: highly supinated, + 12: highly pronated) [39]. The reported intraclass correlation coefficient (ICC) for PFPS was high, at 0.88–0.97 and the inter-rater reliability (ICC) was 0.79–0.88 [9]. Before measuring the participants, the ankles and feet were observed by raising the pants sufficiently to the knees, walking a few steps in place, and then maintaining the posture until the measurements were taken.

**Fig. 2:**
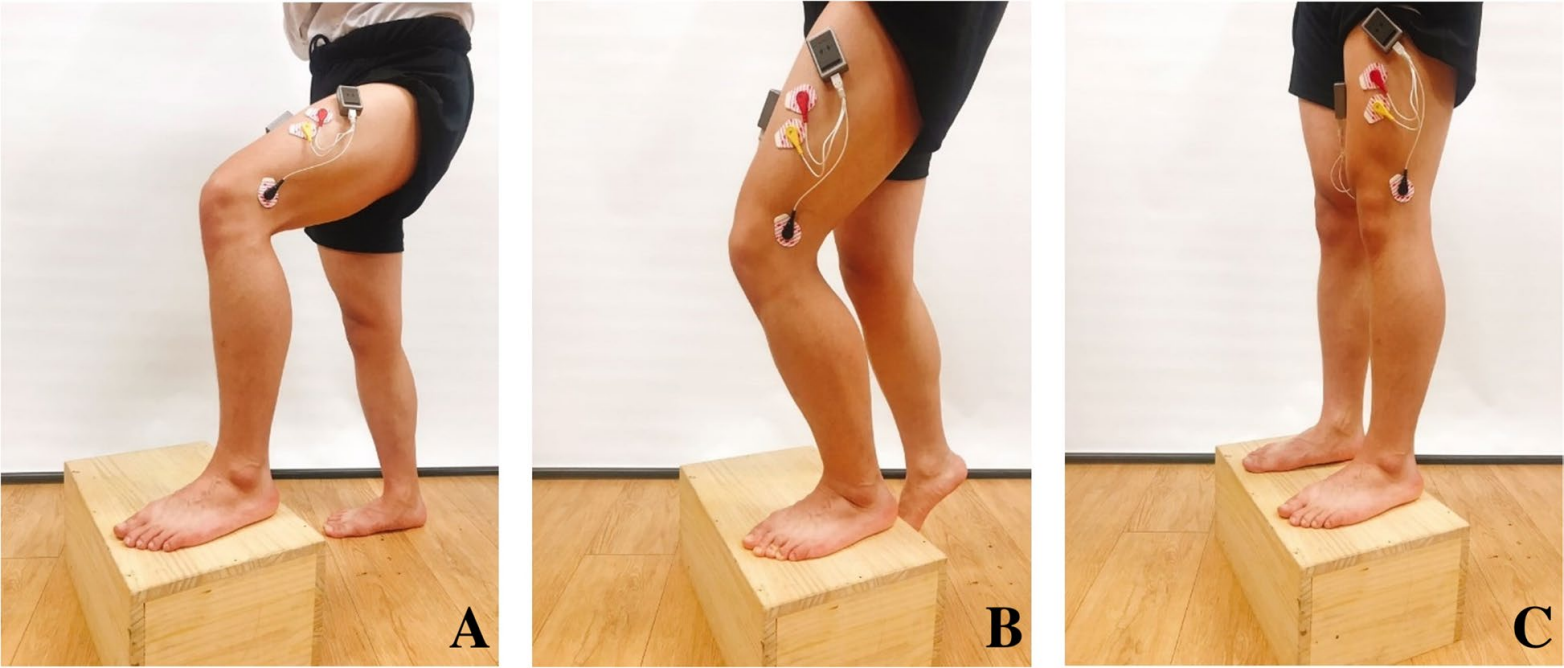
Vastus medialis/vastus lateralis VM/VL muscle activities ratio. **A** Before stair ascent. **B** Ascending a stair with the affected leg. **C** After stair ascent. The maximum value is the ratio of the root mean squares of the vastus medialis and vastus lateralis while ascending the stair

The muscle activity ratio was used to determine the activity ratio of VM to VL. To measure the muscle activity of the VM and VL, four surface electromyography (sEMG) instruments (2EM) were used in integrated motion analysis equipment (4D-MT, Relive, Korea, 2016) [40]. Before attaching the surface electrode (2225H, Hurev, Korea, 2017), depilation was performed and foreign substances were removed using an alcohol swab pad. A surface electrode was attached to the center where the muscle contraction was clearly seen in the maximum isometric contraction position (electrode attachment position of the VM muscle: approximately 4 cm superior and medial to the superomedial border of the patella, VL muscle: approximately 10 cm superior to the superolateral border of the patella) [41, 42]. The muscle activity was measured by using the ratio of the maximum value in the root mean square (RMS) of the data obtained during stair ascent (DIY step box, 445X280X205mm [widthXdepthXheight])(Fig. 2) [43, 44].

### Sample size

The sample size was estimated for the primary outcome (NPRS). After the intervention in the experimental group reported by Fukuda et al. [45], the effect size (Cohen’s d), 0.47, was calculated from the pain reduction value and the effect size f (V) was set to 0.24. Using G*power 3.1 (Franz Faul, Universität Kiel, Germany), the power is 0.80 and the group is set to 3 groups, and at least 13 participants should be assigned to one group. A total of 48 were recruited, taking into account dropout (18.75%) from the calculated 39 participants.

### Statistical analysess

All statistical analyses were performed using the IBM SPSS Statistics version 21.0 (IBM Corp., USA). Homogeneity tests were performed using the chi-squared tests and one-way analysis of variance (ANOVA). Descriptive statistics were used for the baseline characteristics of the participants in the three trial arms. The results of pain, function, alignment, and muscle activity ratio were analyzed by two-way repeated-measures ANOVA. When there were interactions between factors (time ×group) in measurement results, one-way ANOVA was performed to assess the differences in the three trial arms according to the measurement time. Significant differences between the three trial arms were analyzed by Bonferroni’s post hoc tests based on the simple main effect.

In addition, the partial eta square (η^2^_p_) was calculated to investigate the effect size according to the intervention method and the effect size was determined using Cohen’s d (Cohen’s criteria: small ≤0.2; moderate = 0.5; large ≥0.8) to compare the effects of the intervention methods on the dependent variables. The statistical significance level (α) for the ANOVA was set to 0.05 and the significance level (α) of the Bonferroni’s post hoc test was set to 0.017.

## Results

**Fig. 3:**
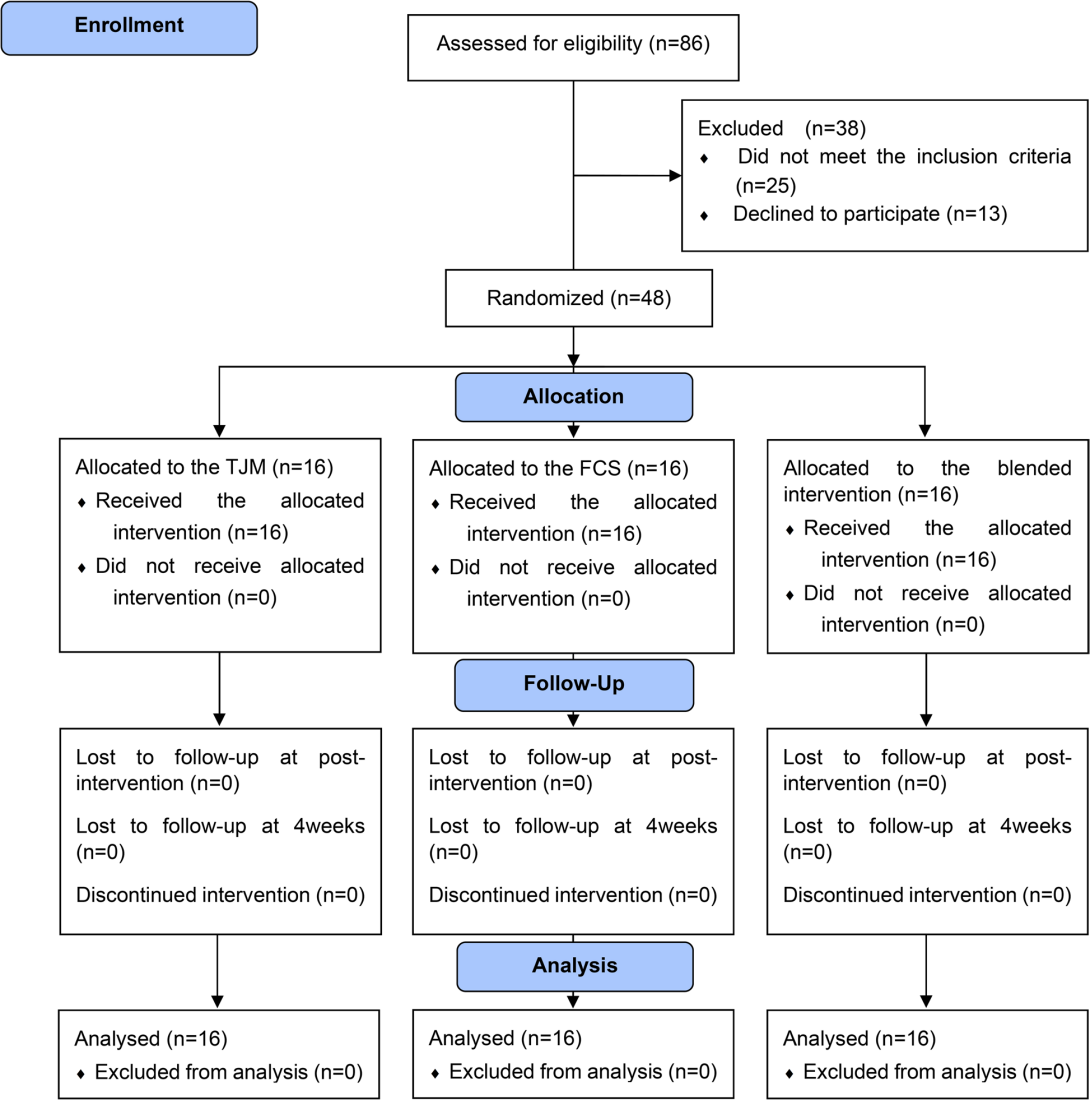
Flow diagram of participant recruitment, allocation, and analysis

From August 2018 to December 2018, forty-eight participants were recruited and received each intervention according to the intervention schedule without dropout. No adverse effects were seen after intervention among participants (Fig. 3). The baseline characteristics of the participants are shown in Table 2. No significant differences were found between groups in general characteristics (*p* > 0.05). The NPRS scores of participants who complained of pain for more than 12 weeks were 4.44 ± 0.73 for TJM, 4.25 ± 0.45 for FCS, and 4.38 ± 0.81 for blended intervention, and there was no significant difference between groups.

**Table 2:** The baseline characteristics of enrolled participants

Variables	TJM (*n* = 16)	FCS (*n* = 16)	Blended Intervention (*n* = 16)	χ^2^/F	*p*-values
Age (Year)	21.38 ± 1.82	21.81 ± 2.29	22.69 ± 2.77	1.320	.277^a^
Sex, female, N (%)	10 (63)	9 (56)	8 (50)	.508	.776^b^
Height (cm)	174.48 ± 8.46	164.75 ± 9.97	178.97 ± 8.03	.894	.416^a^
Weight (kg)	65.38 ± 12.22	63.13 ± 13.07	68.27 ± 10.35	.216	.806^a^
BMI (kg/m^2^)	24.07 ± 2.59	23.30 ± 3.87	23.91 ± 2.26	.317	.682^a^
Foot size (mm)	262.81 ± 15.05	250.00 ± 20.41	266.43 ± 16.73	.386	.872^a^
Affected side, left, N (%)	9 (56)	8 (50)	8 (50)	.167	.920^b^

Values are mean ± SD unless otherwise indicated, *BMI* Body Mass Index, *FCS* Foot Core Strengthening, *TJM* Talonavicular Joint Mobilization

^a^Independent-samples t-tests. There were no differences between groups (*p* > 0.05)

^b^Chi-square tests. There were no differences between groups (*p* > 0.05)

### Pain

**Fig. 4:**
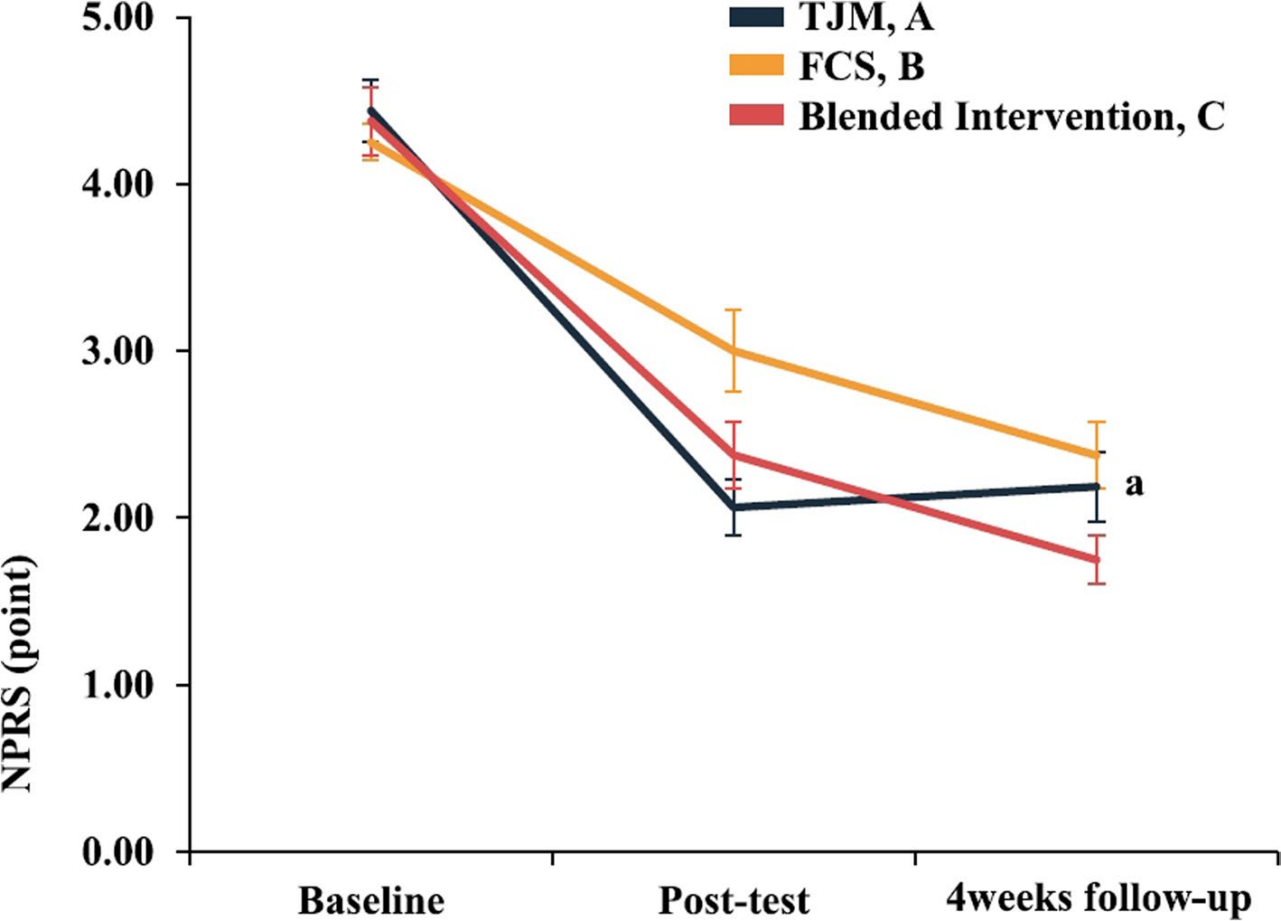
Results for each intervention group according to the evaluation time point. Within-group differences at each point measurement with Bonferroni correction (^a^*P* < 0.017: A > B). Values are provided as means ± standard error of the NPRS score (0–10 points). Abbreviations: FCS, foot core strengthening; NPRS, numeric pain rating scale; TJM, talonavicular joint mobilization

The two-way repeated-measures ANOVA revealed statistically significant NPRS interactions for the evaluation time points in each group (95% confidence interval [CI]: 2.820, 3.138, η^2^_p_ = 0.162). In the Bonferroni’s post-hoc tests, the NPRS was not significantly different post-intervention (*p* > 0.017). At the 4-week follow-up, there was a significant difference in the TJM group compared to the FCS group, but the pain reduction was not maintained (*p* < 0.017)(Fig. 4, Table 3). When each intervention effect was compared numerically using Cohen’s d, the post-intervention effect was the largest in the TJM group (*d* = 3.37). The carryover and preservation effects were the largest in the blended intervention group (*d* = 3.74 / *d* = 1.26)(Fig. 9, Table 4).

**Table 3:** Results for each treatment group according to the evaluation time point

Variables	Talonavicular Joint Mobilization	Foot Core Strengthening	Blended Intervention	Time × Group^b^	Effect size
NPRS^a^					
Baseline	4.44 ± 0.73	4.25 ± 0.45	4.38 ± 0.81	4.355 (2.820, 3.138)^c^	0.162
Post-test	2.06 ± 0.68^d^	3.00 ± 0.97^d^	2.38 ± 0.81^d^		
Follow-up at 4 weeks	2.19 ± 0.83^d^	2.38 ± 0.81^d^	1.75 ± 0.58^d^		
AKPS					
Baseline	74.94 ± 6.01	72.94 ± 6.68	73.50 ± 5.55	4.028 (77.844, 80.545)^c^	0.152
Post-test	81.88 ± 6.34^d^	79.81 ± 5.39^d^	84.31 ± 4.03^d^		
Follow-up at 4 weeks	93.50 ± 5.50	84.31 ± 4.03^d^	85.31 ± 4.03^d^		
DVI^a^					
Baseline	30.35 ± 8.02	31.94 ± 9.22	28.95 ± 5.85	2.576 (20.084, 23.231)^c^	0.103
Post-test	19.33 ± 7.62	17.37 ± 5.79^d^	13.66 ± 8.67^d^		
Follow-up at 4 weeks	23.05 ± 6.77^d^	18.24 ± 7.71^d^	12.03 ± 5.83^d^		
FPI^a^					
Baseline	6.94 ± 1.65	6.56 ± 2.16	6.50 ± 1.55	3.407 (4.479, 5.035)^c^	0.131
Post-test	3.25 ± 1.24	4.50 ± 0.97^d^	4.06 ± 1.00^d^		
Follow-up at 4 weeks	3.13 ± 0.81	4.69 ± 1.35^d^	3.19 ± 1.42^d^		
VM/VL ratio					
Baseline	0.88 ± 0.27	0.87 ± 0.27	0.89 ± 0.09	3.568 (0.977, 1.090)^c^	0.137
Post-test	1.10 ± 0.28	1.06 ± 0.25^d^	1.20 ± 0.14^d^		
Follow-up at 4 weeks	1.00 ± 0.22^d^	1.02 ± 0.32	1.28 ± 0.17^d^		

Values are mean ± SD unless otherwise indicated, *AKPS* Anterior Knee Pain Scale, *DVI* Dynamic Valgus Index, *FPI* Foot Posture Index, *NPRS* Numeric Pain Rating Scale, *VL* Vastus Lateralis, *VM* Vastus Medialis

^a^Lower scores are better

^b^F-value (95% confidence interval)

^c^Time × group interaction effect

^d^Compared to baseline, Effect size = partial eta squared (η^2^_p_)

**Table 4:** Comparisons of the effect size for each intervention method using Cohen’s d

Variables	Talonavicular Joint Mobilization		Foot Core Strengthening		Blended Intervention	
	Pi^a^	Ce^b^	Pi^a^	Ce^b^	Pi^a^	Ce^b^
NPRS	3.37	2.88	1.66	2.88	2.48	3.74
AKPS	1.12	0.68	1.13	1.22	2.23	2.42
DVI	1.41	0.98	1.89	1.61	2.07	2.90
FPI	2.53	2.93	1.23	1.04	1.87	2.23
VM/VL ratio	0.78	0.47	0.74	0.50	2.69	2.93

*AKPS* Anterior Knee Pain Scale, *DVI* Dynamic Valgus Index, *FPI* Foot Posture Index, *NPRS* Numeric Pain Rating Scale, *VL* Vastus Lateralis, *VM* Vastus Medialis

^a^Post-intervention effect: post-test minus baseline

^b^Carryover effect: follow-up at 4 weeks minus baseline

### Lower extremity function

**Fig. 5:**
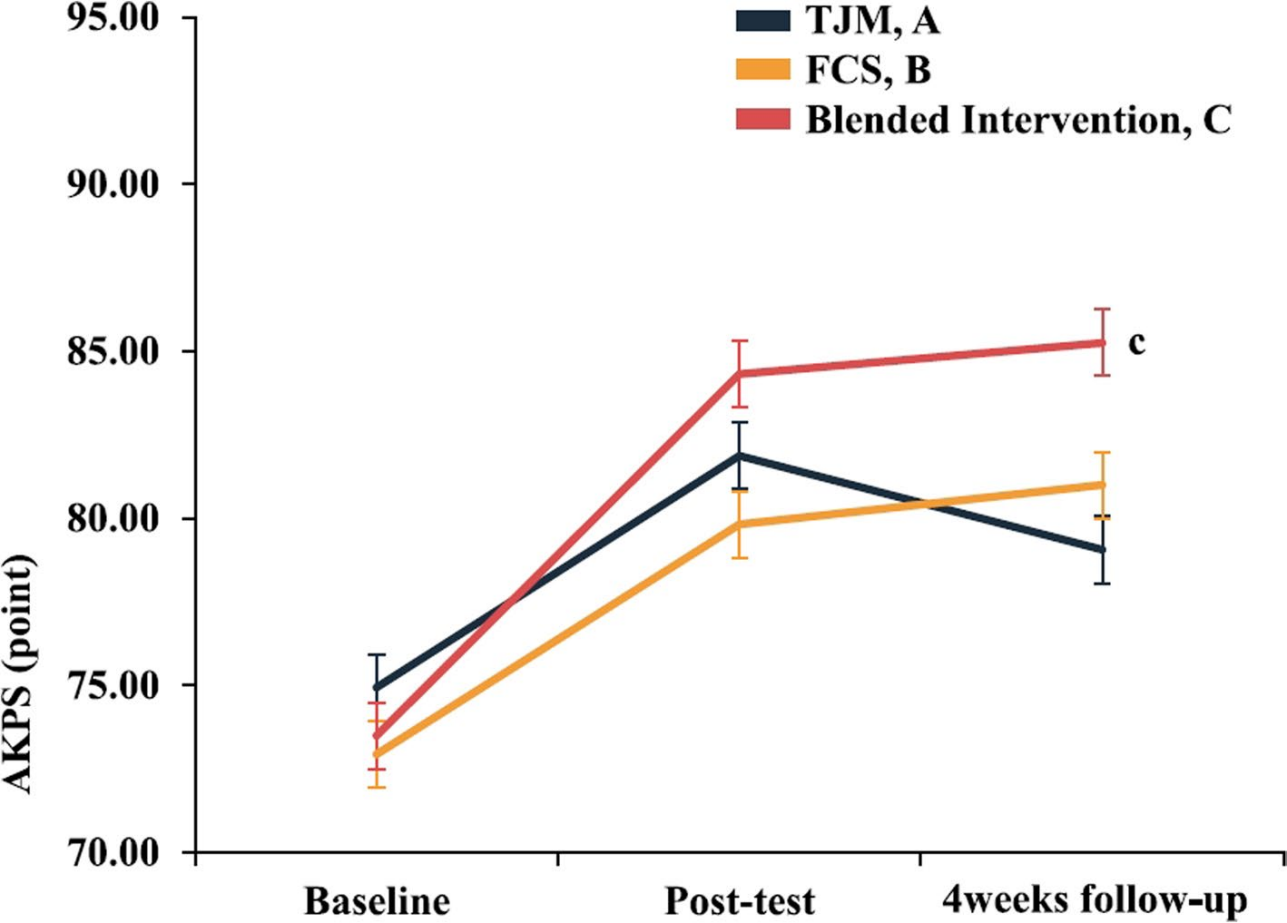
Results for each intervention group according to the evaluation time point. Each-group differences at each point measurement with Bonferroni correction (^c^*P* < 0.017: A < C). alues are mean ± standard error of the AKPS score (0–100 points). Abbreviations: AKPS, anterior knee pain scale; FCS, foot core strengthening; TJM, talonavicular joint mobilization

The two-way repeated-measures ANOVA revealed statistically significant AKPS interactions for evaluation time points for each group (95% CI: 77.844, 80.545, η^2^_p_ = 0.152). In the Bonferroni’s post-hoc test, the AKPS was not significantly different immediately post-intervention (*p* > 0.017); however, at the 4-week follow-up, the blended intervention group showed greater improvement than that in the TJM group (*p* < 0.017)(Fig. 5, Table 3). When each intervention effect was compared numerically using Cohen’s d, the post-intervention effect was largest in the blended intervention group (*d* = 2.23). The carryover and preservation effects were largest in the blended intervention group (*d* = 2.42 / *d* = 0.19)(Fig. 9, Table 4).

### Valgus knee

**Fig. 6:**
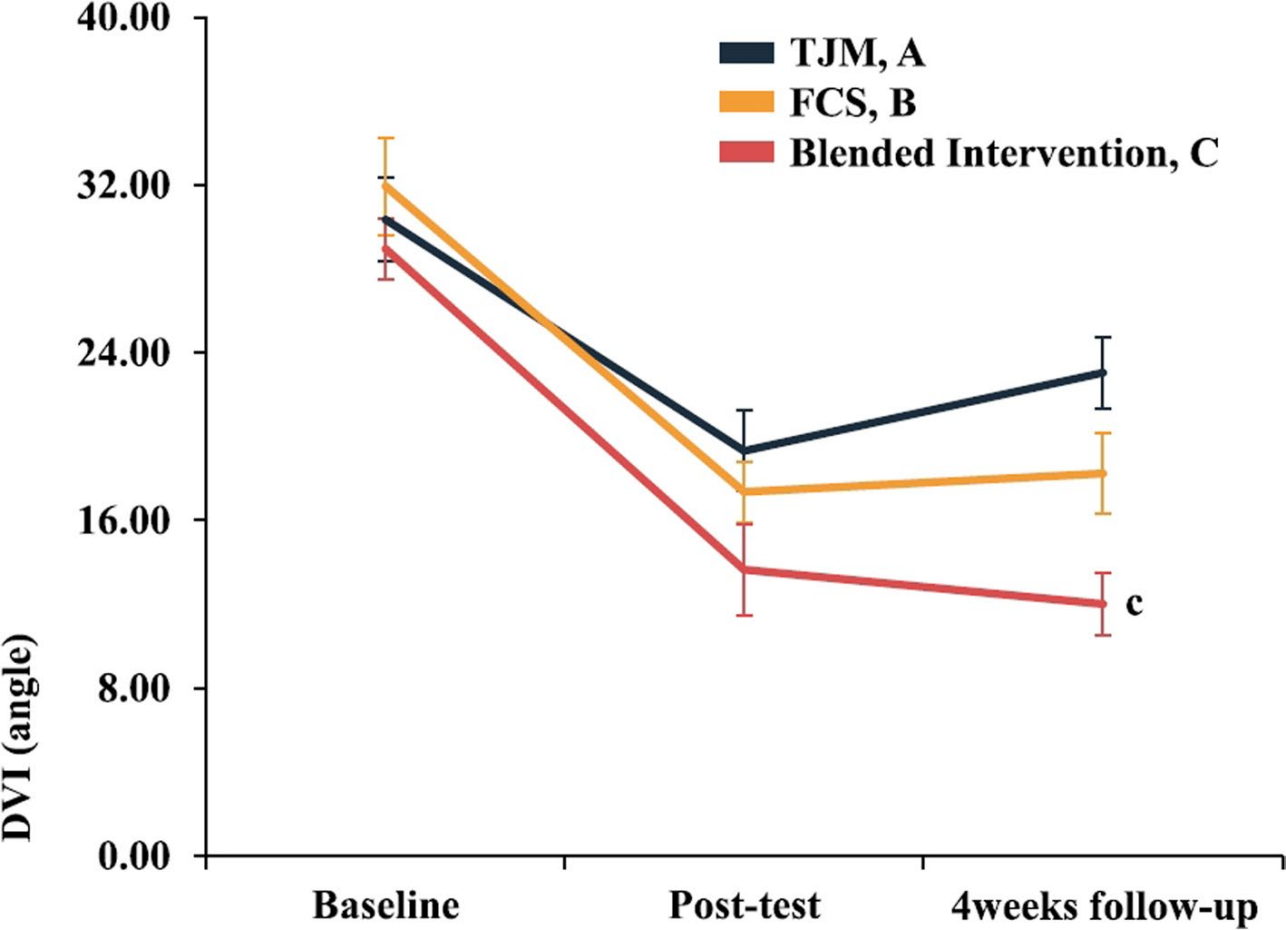
Results for each treatment group according to the evaluation time point. Each-group differences at each point measurement with Bonferroni correction (^c^*P* < 0.017: A < C). Values are mean ± standard error of the AKPS score (0–100 points). Abbreviations: DVI, dynamic valgus index; FCS, foot core strengthening; TJM, talonavicular joint mobilization

The two-way repeated-measures ANOVA revealed statistically significant DVI interaction for the evaluation time points for each group (95% CI: 20.084, 23.231, η^2^_p_ = 0.103). In the Bonferroni’s post-hoc tests, DVI was not significantly different immediately post-intervention (*p* > 0.017); however, at the 4-week follow-up, the blended intervention group showed greater improvement than that in the TJM group (*p* < 0.017)(Fig. 6, Table 3). When each intervention effect was compared numerically using Cohen’s d, the post-intervention effect was largest in the blended intervention group (*d* = 2.07). The carryover and preservation effects were largest in the blended intervention group (*d* = 2.90 / *d* = 0.83)(Fig. 9, Table 4).

### Foot posture

**Fig. 7:**
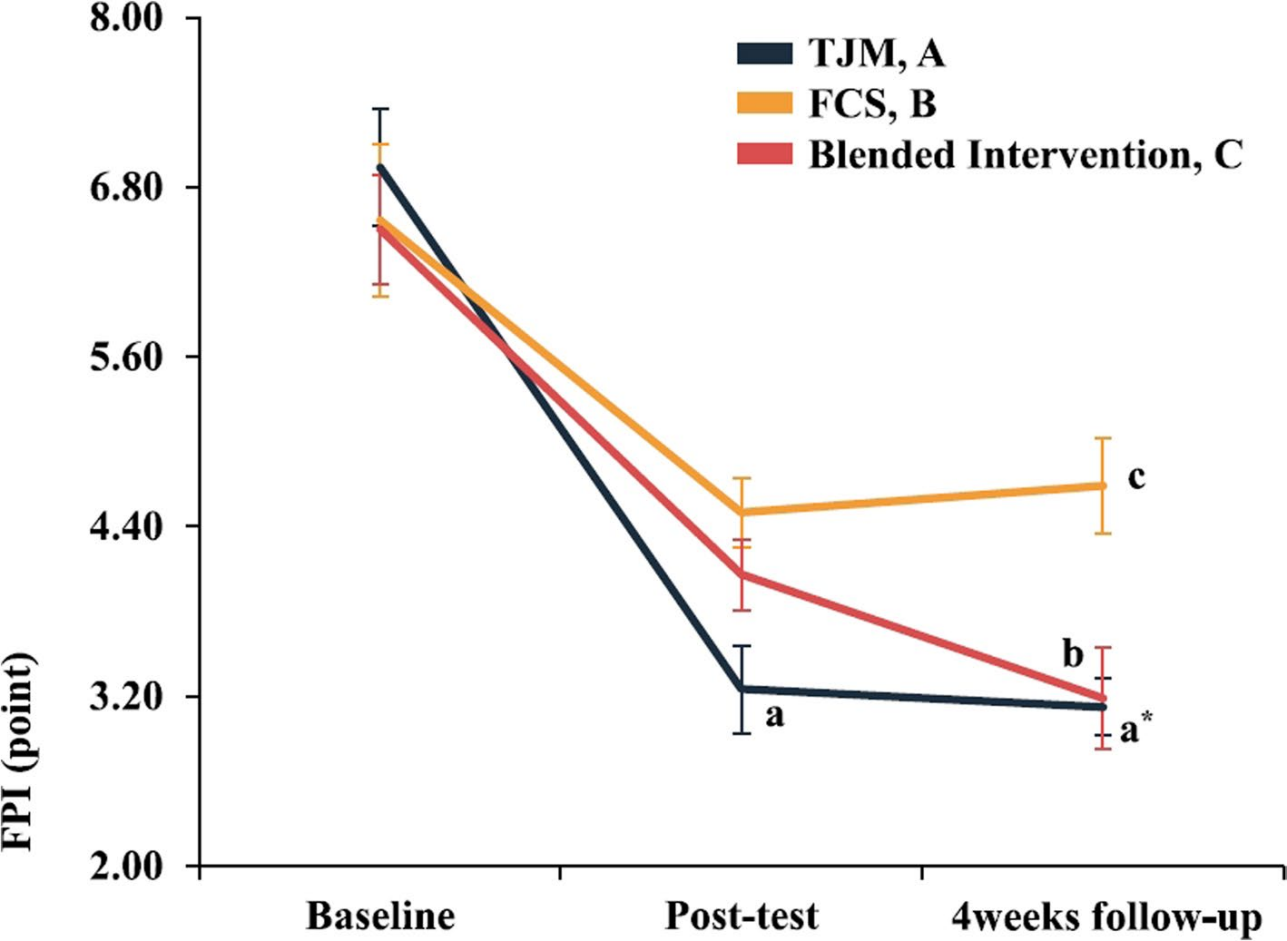
Results for each treatment group according to the evaluation time point. Each-group differences at each point measurement with Bonferroni correction (^a^*P* < 0.017: A > B, ^b^*P* < 0.017: B < C, ^a*^P < 0.017: A > B). Values are mean ± standard error of the AKPS score (0–100 points). Abbreviations: FCS, foot core strengthening; FPI, foot posture index; TJM, talonavicular joint mobilization

The two-way repeated-measures ANOVA revealed statistically significant FPI interactions for the evaluation time points in each group (95% CI: 4.479, 5.035, η^2^_p_ = 0.131). In the Bonferroni’s post-hoc test, the TJM group showed greater improvement than that in the FCS group immediately post-intervention (*p* < 0.017), while the TJM and blended intervention groups showed greater improvements than that in the FCS group at the 4-week follow-up (*p* < 0.017)(Fig. 7, Table 3). When each intervention effect was compared numerically using Cohen’s d, the post-intervention effect was largest in the TJM group (*d* = 2.53). The carryover and preservation effects were largest in the TJM group (*d* = 2.93 / *d* = 0.41)(Fig. 9, Table 4).

### Muscle activity ratio

**Fig. 8:**
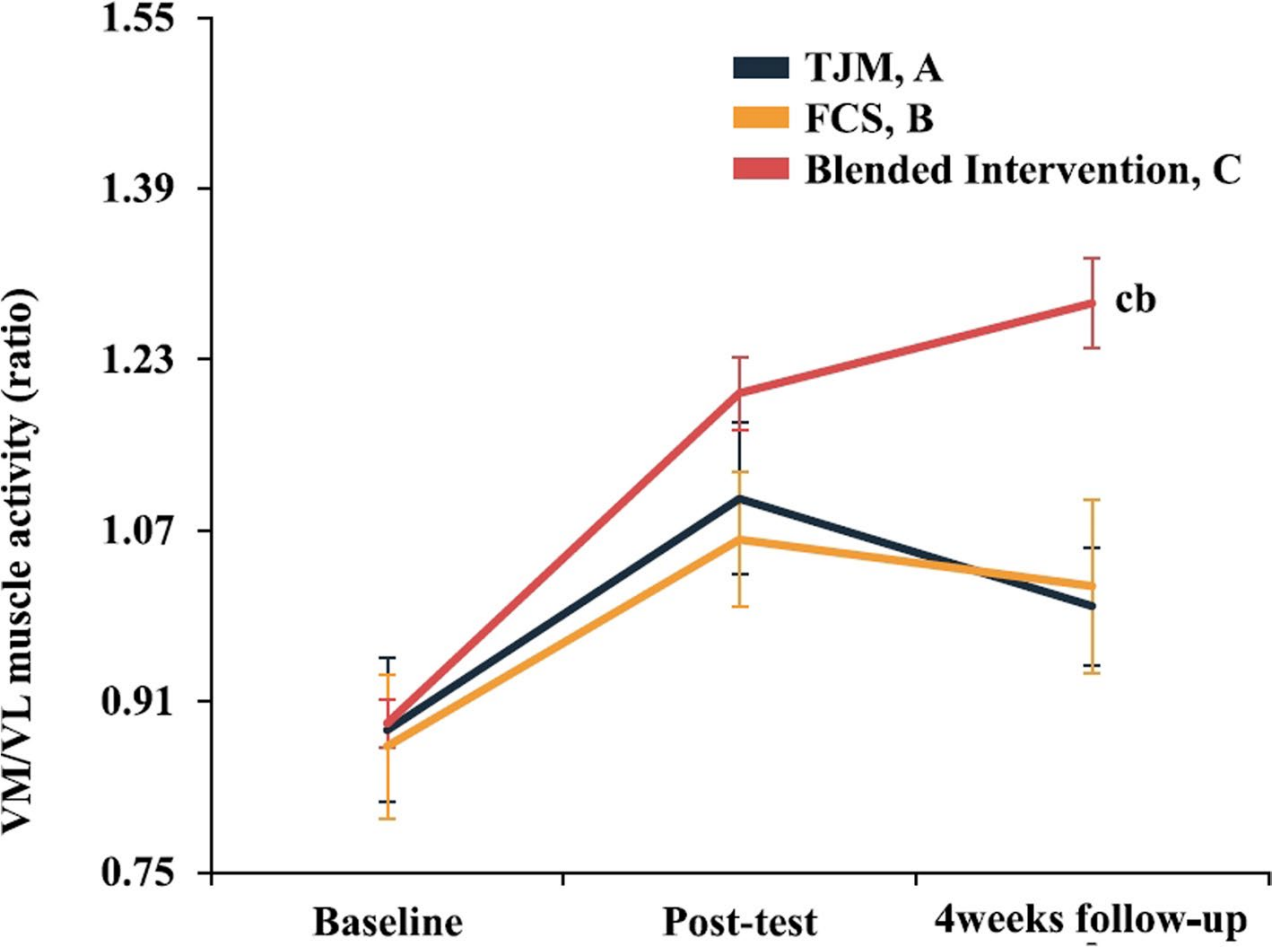
Results for each treatment group according to the evaluation time point. Each-group differences at each point measurement with Bonferroni correction (^b^*P* < 0.017: B < C, ^c^*P* < 0.017: A < C). Values are mean ± standard error of the AKPS score (0–100 points). Abbreviations: FCS, foot core strengthening; TJM, talonavicular joint mobilization; VL, vastus lateralis; VM, vastus medialis

**Fig. 9:**
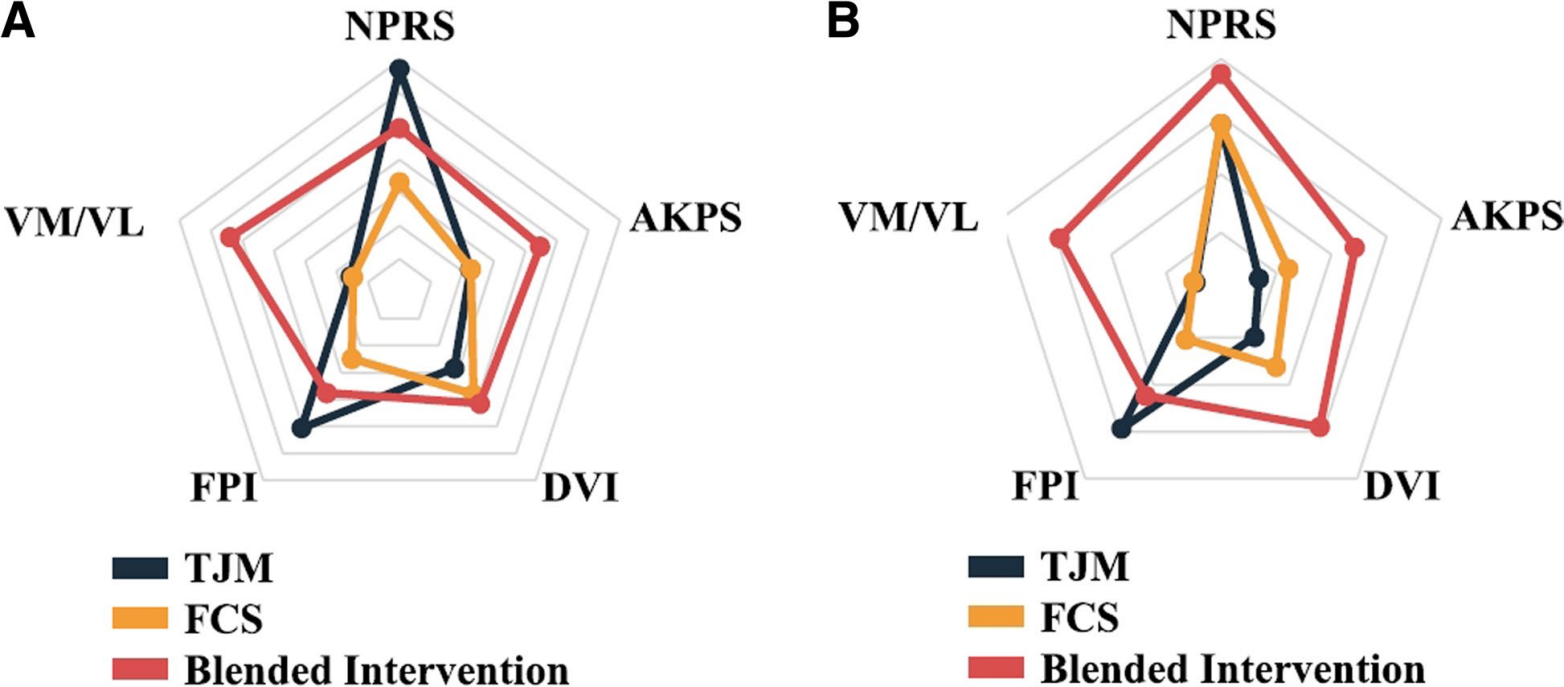
Comparisons of the effects of intervention methods based on Cohen’s d effect sizes. Values are the effect sizes of the intervention methods using Cohen’s d. **A**: Post-intervention effect: post-test minus baseline. **B**: Carryover effect: follow-up at 4 weeks minus baseline. Abbreviations: AKPS, anterior knee pain scale; DVI, dynamic valgus index; FCS, foot core strengthening; FPI, foot posture index; NPRS, numeric pain rating scale; TJM, talonavicular joint mobilization; VL, vastus lateralis; VM, vastus medialis

The two-way repeated-measures ANOVA revealed statistically significant VM/VL ratio interactions for the evaluation time points in each group (95% CI: 0.977, 1.090, η^2^_p_ = 0.137). In the Bonferroni’s post-hoc test, the VM/VL ratio was not significantly different immediately post-intervention (*p* > 0.017); however, at the 4-week follow-up, the blended intervention group showed a greater improvement than those in the FCS and TJM groups (*p* < 0.017)(Fig. 8, Table 3). When each intervention effect was compared numerically using Cohen’s d, the post-intervention effect was largest in the blended intervention group (*d* = 2.69). The carryover and preservation effects were largest in the blended intervention group (*d* = 2.93 / *d* = 0.24)(Fig. 9, Table 4).

## Discussion

Previous studies on PFPS interventions have shown improvement in function and pain mainly through the interventions aimed at the knee or hip joints [2, 6, 46, 47]. However, while the effects of studies on PFPS intervention remain controversial, biomechanical changes by pronated foot, an intrinsic risk factor, can stress the patellofemoral joints and cause patellofemoral pain [24]. This investigated the effects of structural and functional changes on patellofemoral pain following the application of TJM, FCS, and blended interventions in those in their 20s with high PFPS prevalence.

The results of this study showed a significant interaction effect in all groups (*p* < 0.05). TJM had a post-intervention effect on pain and foot posture, while those of the blended intervention were significantly greater than other groups for all dependent variables.

The NPRS and AKPS were clinically significant based on the MCID reported in previous studies [35]. In the case of the NPRS, the TJM group (2.38 points, 2.25 points) and the blended intervention group (2 points, 2.63 points) showed clinically significant differences between the mean values at baseline at post-test and the 4 weeks follow-up. In the case of the AKPS, a significant difference was observed between the blended intervention and TJM groups at the 4-week follow-up. However, although the mean value, 11.75 points, was statistically significant, it was not considered to be clinically meaningful.

However, this study was significant even though the interventions were performed on the foot, which is the distal factor of PFPS, and this is the first study to assess the effects of a physical therapy intervention to the talonavicular joint. The carryover effect of TJM was significantly different only in the FPI; however, we observed a post-intervention effect in pain and foot posture. Results of this study can be supported by previous studies that there is a correlation between patellofemoral pain and pronated foot [48]. The physiological evidence is to improve the movement of the navicular to the dorsal direction in the pronated foot. This suggests that joint mobilization at the talonavicular joint can help activate the muscles around the joints by stimulating mechanoreceptors distributed in the joint and muscle [19, 20, 49, 50]. In addition, the effects of the blended TJM and FCS intervention were significantly greater than those for the other groups at 4 weeks post-intervention. Previous studies showed that FCS, a typical exercise therapy for the pronated foot with flat or lost MLA, can improve the performance of the intrinsic muscles of the foot to improve arch support when joint mobilization and exercise were performed together, with improved intervention and preservation effects [29, 30]. Also, Neal et al. [51], reported that the risk factor for patellofemoral pain was navicular drop in a systematic review. Therefore, considering that FCS is effective in the improvement of navicular drop [18], it is thought to have contributed in part to patellofemoral pain. These findings may support the results of the present study, as exercise-directed interventions under the guidance of a therapist are effective in reducing pain and improving function [52–54].

To resolve the underlying factors of patellofemoral pain, we considered the reduction of pain and improvement of function due to structural changes rather than symptom-based treatment. Based on the fact that the FPI is significantly correlated with the functional state of the lower extremity, we assumed that interventions aimed at the pronated foot would be affected [55]. Barton and Menz et al. reported showed significant improvement in functional changes in foot orthosis, and in the pronated foot during single-leg squat based on the FPI results in 52 patients with patellofemoral pain [56]. The navicular was proposed to have suppressed hypermobility in the plantar direction. Patients with patellofemoral pain syndrome showed a significant difference in dynamic Q-angle and not static Q-angle, following exercise therapy (*p* < 0.01) [57]. This finding may support the DVI results of the present study. This result may be due to differences in muscle activation patterns, which is supported by the PFPS of the VM measured by three items (insertion level, fiber angle, and volume) (*p* < 0.05) [58]. Therefore, the PFPS showed more muscle activity of the VL than the VM, which may have more influence on dynamic Q-angle than static Q-angle.

The pronated foot, which is associated with patellofemoral pain, is a structural change that modulates navicular motility, realigns the rotated femur and tibia, and increases VM muscle activity. Therefore, the clinical significance of this study suggests that management of the talonavicular joint, not just an intervention program related to the knee or hip joint, is necessary to reduce pain and improve function for individuals with PFPS. The limitations in this study design were the short durations of the intervention (12 sessions) and follow-up period (4 weeks). It was also a single-center study, not a multicenter study, and young adults were recruited from the university. In the age-related study on the incidence of PFPS, the rate was 2.63 times higher in those in their 50s than in their 20s [59], so a study design that includes middle-aged (40–60 years old) participants is needed. And in the protocol, the pronated foot intervention did not include the management of the subtalar joints.

## Conclusions

The results of this study suggested that talonavicular joint mobilization is effective for immediate control of patellofemoral pain and foot posture. In addition, in the carryover effect, it was found that the blended intervention has a positive effect on reduced dynamic knee valgus, increased vastus medialis muscle activity to vastus lateralis, and controlled pain. Therefore, talonavicular joint mobilization affects the structural and functional factors of patellofemoral pain, and foot core strengthening is effective for postural control.

## Supplementary Information

**Additional file 1.** Talonavicular joint.

**Additional file 2.** Foot core strengthening.

## Data Availability

The datasets used and/or analyzed during the current study are available from the corresponding author on reasonable request.

## Abbreviations

AKPS: Anterior knee pain scale
ANOVA: Analysis of variance
DVI: Dynamic valgus index
FCS: Foot core strengthening
FPI: Foot posture index
FPPA: Frontal plane projection angle
ICC: Intraclass correlation coefficient
MCID: Minimum clinically importance difference
MLA: Medial longitudinal arch
NPRS: Numeric pain rating scale
PFPS: Patellofemoral pain syndrome
TJM: Talonavicular joint mobilization
VL: Vastus lateralis
VM: Vastus medialis
